# Post exposure prophylaxis following occupational exposure to HIV: a survey of health care workers in Mbeya, Tanzania, 2009-2010

**DOI:** 10.11604/pamj.2015.21.32.4996

**Published:** 2015-05-15

**Authors:** Marcelina John Mponela, Obinna Ositadimma Oleribe, Ahmed Abade, Gideon Kwesigabo

**Affiliations:** 1 Tanzania Field Epidemiology and Laboratory Training Program (TFELTP), Tanzania; 2 Muhimbili University of Health and Allied Sciences (MUHAS), Tanzania; 3 Excellence & Friends Management Care Center, Abuja, Nigeria

**Keywords:** Post exposure prophylaxis, health care workers, occupational exposure, HIV

## Abstract

**Introduction:**

Approximately, 1,000 HIV infections are transmitted annually to health care workers (HCWs) worldwide from occupational exposures. Tanzania HCWs experience one to nine needle stick injuries (NSIs) per year, yet the use of post-exposure prophylaxis (PEP) is largely undocumented. We assessed factors influencing use of PEP among HCWs following occupational exposure to HIV.

**Methods:**

A cross-sectional study was conducted in Mbeya Referral Hospital, Mbozi and Mbarali District Hospitals from December 2009 to January 2010 with a sample size of 360 HCWs. Participants were randomly selected from a list of eligible HCWs in Mbeya hospital and all eligible HCWs were enrolled in the two District Hospitals. Information regarding risk of exposure to body fluids and NSIs were collected using a questionnaire. Logistic regression was done to identify predictors for PEP use using Epi Info 3.5.1 at 95% confidence interval.

**Results:**

Of 291 HCWs who participated in the study, 35.1% (102/291) were exposed to NSIs and body fluids, with NSIs accounting for 62.9% (64/102). Exposure was highest among medical attendants 38.8% (33/85). Out of exposed HCWs, (22.5% (23/102) used HIV PEP with females more likely to use PEP than males. Reporting of exposures (OR=21.1, CI: 3.85-115.62) and having PEP knowledge (OR =6.5, CI: 1.78-23.99) were significantly associated with using PEP.

**Conclusion:**

Despite the observed rate of occupational exposure to HCWs in Tanzania, use of PEP is still low. Effective prevention from HIV infection at work places is required through proper training of HCWs on PEP with emphasis on timely reporting of exposures.

## Introduction

HIV/AIDS and other blood- borne infectious diseases continue to be a major public health problem in developing countries including Tanzania. As the prevalence of the HIV infection continues to rise, HCWs in all geographical regions can expect an increasing frequency in the number or incidences of contacts to patients with HIV/AIDS hence putting them at risk of contracting the infection due to their occupation [1, 2]. Tanzania has an overall HIV prevalence of 5.1% with Njombe, Iringa and Mbeya regions bearing the greatest burden [3].

Globally, it is estimated that up to three million percutaneous exposures occur among HCWs; 90% occurring in least developed countries [4, 5]. Because of these exposures, up to 1000 HIV infections are transmitted annually to HCWs. In Tanzania, HCWs experience between one to nine needle stick injuries per year [6]. Occupational HIV infections in HCWs can be prevented by timely administering Post Exposure Prophylaxis (PEP) [7]. PEP can reduce the risk of HIV infection by up to 81% if properly used [8]. Despite the high HIV prevalence in Tanzania which places HCWs at a high risk of contracting HIV at their work places there is little record of occupational exposure to HIV. There is lack of proper documentation showing the extent of PEP use among HCWs following exposure. This study assessed PEP use and its associated factors among HCWs in Mbeya region

## Methods

### Study design and setting

We conducted a cross sectional study in Mbeya Referral hospital, Mbozi and Mbarali Districts Hospitals from December 2009 to January 2010. Mbeya region comprises of eight districts with an estimated population of 258,000 and a 9.2% HIV prevalence; the second highest prevalence in Tanzania. The region has several health facilities including eight district hospitals, one regional hospital and one referral hospital.

### Study population

All HCWs at risk of exposure to infectious materials like blood, tissue, specific body fluids and equipment or environmental surfaces potentially contaminated with HIV were eligible for this study. These included clinicians, dental personnel, laboratory personnel, nurses, medical attendants and cleaners.

### Sample size and sampling

A sample size of 360 HCWs was obtained by using 52.9% prevalence of needle stick injuries in Tanzania [9]. Mbeya Referral Hospital was purposively selected because of its high number of HCWs with different specialties while the two district hospitals were selected randomly from a list of eight district hospitals. All eligible HCWs within selected district hospitals were enrolled due to the low numbers of workers while those in Mbeya Referral Hospital were randomly selected from a sample frame of all eligible HCWs in the hospital.

### Data collection and analysis

Data was collected using a semi- structured questionnaire and analysis was done using Epi Info version 3.5.1 software. Descriptive statistics were used to determine frequency of social demographic factors and chi-square test was employed to assess association among variables. Logistic regression was conducted to identify predictors for use of PEP at 95% confidence interval (CI).

### Operational definitions

*Occupational exposure* was defined as any percutaneous injury (e.g. a needle stick prick or cut with a sharp object) or contact of mucous membrane or non-intact skin (e.g. exposed skin that is chapped, abraded, or afflicted with dermatitis) with blood, tissue, or other body fluids that are potentially infectious occurring at the workplace.

*PEP Use* was defined as the timely provision of ARV medication following an exposure to potentially infected blood or other body fluids in order to minimize the risk of acquiring infection; consistent with the Tanzania national infection prevention and control guidelines for Health care service. The drugs should be provided within 72 hours; recommended drugs for low risk HIV exposures are a combination of Zidovudine (AZT) and Lamivudine (3TC) while for high risk exposures triple therapy should be used i.e. Zidovudine (AZT) +Lamivudine (3TC) and Efavirenz (EFV).

*Occupational PEP* (sometimes called “oPEP”), taken when someone working in a healthcare setting is potentially exposed to material infected with HIV

*HIV PEP Knowledge* was defined as what was known by the health care worker regarding PEP use and all procedures to follow once a health worker is exposed as stipulated in the National infection prevention and control guidelines. Level of knowledge was assessed by asking five questions. HCWs correctly answered at least 3 questions then had good knowledge of PEP. A total score of 0-59 was graded as having “low knowledge” while 60-100 was graded as having “high knowledge.

### Ethical clearance

Ethical clearance was obtained from Muhimbili University of Health and Allied Sciences (MUHAS) for the entire study. Permission to conduct the study was obtained from Mbeya Region Medical officer (RMO), District Medical Officers (DMOs) and the Director of Mbeya Referral hospital. Informed consent was obtained from participants.

## Results

Of the 360 eligible HCWs, 291 participated in the study. There were 206 (70.8%) females respondents. The respondents mean age was 41.6 years (SD 9.82) ranging from 21-64 years with 58% (169/291) older than 40 years. Majority of the respondents (81%; 206/291) had been married at some point in life, more than half (53.2%; 155/291) had higher education (college and university) and most were nurses (41.6%; 121/291) and medical attendants (29.2%; 85/291) as shown in (Table 1).

**Table 1:** Socio demographic characteristics of respondent in Mbeya, 2009-2010

Characteristics	Number N=291	Percent %
**Hospital**		
Mbeya Referral	191	65.6
Mbozi District	69	23.7
Mbarali District	31	10.7
**Sex**		
Female	206	70.8
Male	85	29.2
**Age group ( years)**		
21-30	56	19.2
31-40	66	22.7
41-50	115	39.5
51+	54	18.6
**Marital status**		
Ever married	236	81.1
Never married	55	18.9
**Education level**		
Primary	86	29.6
Secondary	50	17.2
Tertiary	155	53.2
**Cadre**		
Nurses	121	41.6
Medical attendant	85	29.2
Clinicians	50	17.2
Lab. technicians	18	6.2
Cleaners	12	14.1
Dental personnel	5	1.7
**Working experience (years)**		
0-1	42	14.4
2-10	81	27.8
>10	168	57.7

### Occupational exposure and management

**Figure 1:**
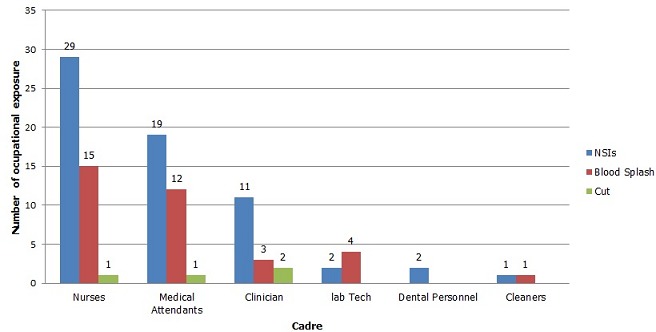
Source of HIV PEP information among HCWs, Mbeya

Out of 291 respondents, 35.1% (102/291) had experienced occupational exposure to HIV in the last twelve months. The most exposed cadres were medical attendants 38.8% (33/85) and nurses 36.4% (44/121) respectively as shown in (Table 2). The most frequent exposure was needle stick injuries (NSIs) with 64 episodes (Figure 1). On average, 0.2 NSIs occurred per HCW per year. Out of 102 HCWs who experienced occupational exposure, 47 (46.1%) reported the exposures to their supervisors. Of those exposed, 25.5% (26/102) of the exposures were from HIV positive patients and risk assessment was conducted for only nine of those who reported the exposures to their supervisor.

**Table 2:** Occupation exposure by Cadre, Mbeya, 2009-2010

*Cadre*	Exposed n=102		Total (%)
	Yes (%)	No (%)	
Nurse	44 (36.4)	77 (63.6)	121 (41.6)
Medical attendants	33 (38.8)	52 (61.2)	85 (29.2)
clinicians	15 (30.0)	35 (70.0)	50 (17.2)
Lab tech	6 (33.3)	12 (66.7)	18 (6.2)
Dental personnel	2 (40.0)	3 (60.0)	5 (1.7)
Cleaners	2 (16.7)	10 (83.3)	12 (4.1)
**Total**	**102 (35.1)**	**189 (64.9)**	**291 (100)**

### PEP use

Out of 102 HCWs with occupational HIV exposure, only 23 (22.5%) used PEP. Only seven (30.4%) of the latter knew the type of drugs given following the exposure. Nurses 52.2% (12/23) and medical attendants 39.1% (9/23) were the most likely groups to use PEP. All HCWs who used PEP were given the drugs within 72 hours, but only 14 of them completed the four weeks dose.

### Knowledge of PEP among health care workers

Of the total respondents, 80.4% (234/291) had ever heard about HIV PEP. Of those who heard about PEP, 56.8% (133/291) had actually got PEP training most of whom were clinicians (27.8%, 37/133) while medical attendants were the least (13.5%, 18/133). On assessing level of HIV PEP knowledge, 62.2% (181/291) had low HIV PEP knowledge with all 12 cleaners and most medical attendants (90.6%, 77/85) having low knowledge. Of the total respondents, 41.9% (122/291) revealed the availability of a focal person for PEP services provision. PEP guidelines were available in all three hospitals.

### Factors influencing use of HIV PEP among HCWs

In bivariate analysis, being female (cOR: 7.9, CI: 1.01-62.82), reporting exposures (cOR: 21.4, CI: 4.66-98.30), knowing HIV status of the source patient (cOR: 4.08, CI: 1.41-13.24) and PEP knowledge (cOR: 4.9, CI: 1.80-13.51) were associated with PEP use. However following multiple logistic regression in order to control for potential confounders, only three factors were statistically significant as shown in Table 3.

**Table 3:** Predictors of PEP use among Healthcare Workers in Mbeya Region- 2009-2010

Variable	Number	Crude OR	95% CI	Adjusted OR	95% CI
***Sex***					
Female	80	7.9	1.01-62.82	22.41	2.25-223.56
Male	22	1		1	
***Age (years)***					
≤ 30	18	1			
≥ 30	84	0.64	0.17-2.44		
***Type of injury***					
Percutaneous	68	2.9	0.90-9.37		
Splashed by blood	34	1			
***Times exposed***					
Once	72	3.5	0.94- 12.49		
>once	30	1			
***Reporting of exposure***					
Reported	47	21.4	4.66-98.30	21.1	3.85-115.62
Not reported	55	1		1	
***Working department***					
High risk department	32	1.22	10.46-3.27		
Low risk department	70	1			
***HIV status of the source patient***					
Known	55	4.08	1.41-13.24		
Not known	47	1			
**PEP knowledge**					
High knowledge	41	4.9	1.80-13.51	6.54	1.78-23.99
Low knowledge	61	1		1	

High risk department are: maternity wards, Theater, surgical wards and laboratory Low risk departments are other wards apart from those mentioned above Percutaneous injuries include needle stick injuries and cut with the sharp objects

## Discussion

This study assessed the prevalence of PEP use among HCWs in Mbeya region. We found a high incidence of NSIs among healthcare workers and low use of PEP. Reporting of exposures and PEP knowledge were predictors for PEP use. Up to 35% of respondents experienced occupational exposure to HIV. This percentage is lower than that of a study done in Mulago National Referral hospital in Uganda where it was observed that 82.9% of health care workers were exposed [10]. The observed difference might be due to recall bias since respondents had to remember exposures occurred in the past one year. It is also possible that Mulago being a national referral hospital, it is busier, exposing HCWs to a higher risk of exposure. The study has also shown that, the most affected cadres were nurses and medical attendants. These two cadres of HCWs interact closely with patients and are involved in the most hazardous activities like dressing wounds, injecting, cleaning, surgical operations and patients care. Other studies have similar findings [11–13].

Majority of the exposures were needle stick injuries (NSIs). different studies have shown similar result [14–17]. The possible reasons for NSIs to be the most common exposure are; overuse of injections and unnecessary sharps, lack of supplies (disposable syringes, safer needle devices, and sharps-disposal containers), lack of access to and failure to use sharps containers immediately after injection, inadequate or short staffing, recapping of needles after use, lack of engineering controls such as safer needle devices, passing instruments from hand to hand in the operating theatre, lack of awareness of hazard and lack of training as shown in a study done by Wilburn and Lee respectively [18, 19]. On the average, 0.2 NSIs occurred per HCW per year. This average is much smaller than that reported by Wilburn et al, [18, 20] in African, Eastern Mediterranean and Asian populations and Mbaisi, kenya. Another study done in Mwanza showed on the average, a HCW is pricked five times per year [21]. This difference might have been due to differences in knowledge and skills on infection prevention control measures at their respective workplaces and study design used.

Use of PEP among HCWs in developing countries is still low compared to other developed countries [22]. In this study, low use of PEP was observed compared to studies done in other developing countries (Mulago Hospital, Uganda and Thika District, Kenya) some of the reasons being inadequate knowledge on PEP, low accessibility of PEP service and perceiving NSIs as low risk [10, 23–25]

In this study, more than 50% of the exposed HCWs did not report their exposures. HCWs who reported their exposures were more likely to use PEP than those who didn't. Workers may not report these exposures for a number of reasons including; not perceiving the risk of the incident, lack of programs for PEP follow up and prophylaxis or the worker's ignorance about PEP, fear of a possible positive result and its associated stigma among other reasons. Several studies have shown underreporting of occupational exposures in their work places especially in developing countries [5, 26–28]

The study showed that, having high knowledge on PEP was a contributing factor for its use. Although more than 50% of HCWs attended PEP training, 60% of them had low level of PEP knowledge especially among the lower level cadre (medical attendants. Several similar studies have found PEP knowledge among HCWs is still inadequate [29, 30].

A number of limitations should be considered in the interpretation of these findings. Recall bias may have affected reported frequency of occupational exposure events.

## Conclusion

The study showed a low rate of occupational exposure, low reporting rate of exposures together with low use of PEP following occupational exposure to HIV. Reporting of exposures and high level of PEP knowledge were significantly associated with PEP use. Health Care Workers should be trained on PEP regardless of their cadres, PEP guidelines should be provided and followed in all facilities, and bio-hazard should be properly managed and timely reporting of exposures. Hospitals should provide practical steps to reduce the rate of NSIs among HCWs and institute supportive supervision to ensure infection prevention measures are adhered to.
